# The Relationship Between FIB-4 Score and Dynapenia in Older Adults

**DOI:** 10.3390/diagnostics15182323

**Published:** 2025-09-13

**Authors:** Guner Kilic, Ali Karatas, Mehmet Cindoruk, Tarkan Karakan, Derya Kirman, Beril Demir, Suleyman Emre Kocyigit

**Affiliations:** 1 Division of Gastroenterology, Department of Internal Medicine, Faculty of Medicine, Gazi University, Ankara 06560, Turkey; 2 Geriatrics Division, Department of Internal Medicine, Faculty of Medicine, Balıkesir University, Balıkesir 10145, Turkey

**Keywords:** FIB-4, dynapenia, ageing, comprehensive geriatric assessment

## Abstract

**Background/Objectives:** We evaluated whether fibrosis-4 (FIB-4) is associated with dynapenia and functional status in adults ≥65 years, and its value as a geriatric screening tool. **Methods**: In this cross-sectional study (July 2023–July 2025), 537 outpatients aged ≥65 years were evaluated. FIB-4 was calculated by the standard formula; a high-risk threshold of ≥2.0 was applied for age ≥65. Participants were compared as low- and high-FIB-4. Functional status was assessed by Basic Activities of Daily Living (ADLs), instrumental ADLs, and gait and balance tests. Dynapenia was defined as low muscle strength. Comorbidities, geriatric syndromes, and laboratory findings were recorded. **Results:** The high-FIB-4 group was older (78.6 ± 6.0 vs. 75.6 ± 5.8 years), had fewer women, and had lower BMI. POMA and instrumental ADLs scores were lower in the high-FIB-4 group, while basic ADLs and TUG duration did not differ significantly. Low grip strength was more frequent with high FIB-4. In sex-stratified analyses, FIB-4 correlated positively with age and aspartate transaminase (AST), and negatively with platelet count, leukocyte count, handgrip strength, POMA, and instrumental ADLs in women. In regression analysis low grip strength was associated with high FIB-4 score, but this relationship disappeared regardless of confounding factor in older female people. Lower BMI and higher leucocyte count is a risk factor for high FIB-4 score in the dynapenia group. **Conclusions:** FIB-4 reflects not only hepatic fibrosis risk but also geriatric vulnerability linked to dynapenia and functional decline in older adults. With age-adjusted cutoffs, FIB-4 may serve as a practical early-warning screen alongside nutritional and physical-performance assessments.

## 1. Introduction

Liver biopsy remains the gold standard method for assessing fibrosis. However, the procedure is invasive and carries a risk of major complications in 1–3% of cases and a mortality risk of approximately 1 in 10,000 [1,2]. For the assessment of hepatic fibrosis, priority should be given to non-invasive methods that offer high measurement reliability and have proven cost-effectiveness [3]. The fibrosis-4 (FIB-4) score, with easily accessible parameters such as age, aspartate transaminase (AST), alanine transaminase (ALT), and thrombocyte count, is a scoring system that was developed to predict liver fibrosis. It is currently widely used in the evaluation of liver fibrosis, and in the follow-up of chronic liver diseases such as metabolic dysfunction-associated steatotic liver disease (MASLD) and advanced cirrhosis [4]. The FIB-4 score is recommended as one of the first-stage tests in MASLD and the screening of individuals at risk of advanced fibrosis. The FIB-4 was developed as a simple, non-invasive tool to determine the presence of advanced fibrosis, and categorises the fibrosis risk as low (<1.30), uncertain (1.30–2.67), or high (>2.67). The defined threshold values function as critical limits in non-invasive risk evaluation strategies and guide treatment planning with advanced level diagnostic evaluation [5,6,7,8]. However, as age is a component of the FIB-4, the score increases as patients age, even without any change in the other components of the score. Recently published guidelines have emphasized the need to apply the FIB-4 score according to age, and recommend a low-risk threshold value of 2.0 for individuals aged ≥65 years [9].

Sarcopenia is a geriatric syndrome characterized by progressive loss of muscle mass and strength. Dynapenia is defined as low muscle strength. Sarcopenia has negative consequences such as disability, falls, impaired quality of life, and increased mortality [10]. The literature suggests that sarcopenia, whose prevalence increases with age, shares pathophysiological mechanisms with non-alcoholic fatty liver disease (NAFLD), and that a bidirectional relationship may exist between them. Common mechanisms between sarcopenia and NAFLD include insulin resistance, hormonal imbalance, systemic inflammation, myostatin and adiponectin dysregulation, nutritional deficiencies, and physical inactivity [11]. Furthermore, myosteatosis and muscle quality may play important roles in the development of metabolic-related fatty liver disease (MAFLD), even in non-obese elderly individuals [12]. In other words, sarcopenia stands out as a risk factor for MAFLD. Metabolic dysfunction-related fatty liver disease increases with age, and advanced age is a prominent predictor of liver fibrosis [13]. Screening patients with MAFLD, especially older adults, for fibrosis risk using noninvasive methods such as FIB-4 is recommended. The relationship between this score and low grip strength, which shares common pathophysiological mechanisms, is unknown.

Furthermore, the inclusion of chronological age, rather than biological age, in the FIB-4 score appears to be a significant drawback. There are significant doubts about the validity of this score in predicting or screening for liver fibrosis, particularly in geriatric practice. Its reliability in histopathologically detecting liver fibrosis is low [14]. Reasons for this condition may include selection bias and the heterogeneity of elderly individuals. Furthermore, the literature lacks sufficient studies on the reliability of non-invasive screening methods such as the FIB-4 score in older adults with low muscle strength or sarcopenia, which are closely associated with liver fibrosis. The aim of this study was to evaluate the relationship between the FIB-4 score and low muscle strength in individuals aged ≥65 years.

## 2. Materials & Methods

### 2.1. Study Design and Participants

We conducted a single-center, cross-sectional study in the geriatric outpatient clinic of a tertiary care university hospital. Consecutive adults aged ≥65 years who attended routine visits between July 2023 and July 2025 were retrospectively screened for eligibility and enrolled if inclusion criteria were met. Sample size was calculated using the G power test. Considering the effect size as 0.4, power as 0.90, and alpha error as 0.05, the minimum total sample size was calculated as 272.

### 2.2. Eligibility Criteria

Inclusion required age ≥65 years and availability of a same-day clinical assessment and laboratory panel. Exclusion criteria were prespecified to minimize reverse causation and acute confounding: active malignancy; compensated or decompensated liver cirrhosis; alcoholic steatohepatitis; chronic viral hepatitis; end-stage renal failure; severe anemia (hemoglobin < 10 g/dL); recent acute cerebrovascular event; acute infection; immobility precluding performance testing; and incomplete core data. Comorbid diseases including hypertension, diabetes, coronary artery disease, COPD, osteoporosis, dementia were extracted from the electronic record and verified by an experienced geriatrician during the index visit.

The study was conducted in accordance with the Declaration of Helsinki, and the protocol was approved by the Ethics Committee of Balikesir University (approval code: 278) on 5 August 2025. Informed consent for participation was obtained from all subjects involved in the study.

### 2.3. Data Collection and Clinical Evaluation

A record was made for each study participant of sociodemographic characteristics (age, gender, education level, marital status), comorbid diseases (hypertension, diabetes mellitus, coronary artery disease, dementia, Chronic Obstructive Pulmonary Disease (COPD), osteoporosis, etc.) and geriatric syndromes (malnutrition, history of recurrent falls in a year, frailty). Comprehensive geriatric assessment parameters were performed using the Basic Activities of Daily Living (ADLs), the Lawton Instrumental ADLs scale, the Tinetti Performance-Oriented Mobility Assessment (POMA), and the Timed Up and Go (TUG) test duration [15]. Hand grip strength was measured with a Handgrip strength was measured using a calibrated Jamar hand dynamometer (Performance Health, Warrenville, IL, USA).

### 2.4. Calculation of the FIB-4 Score

The FIB-4 score was calculated using the confirmed formula below:

FIB-4 = (Age [years] × AST [U/L])/(Platelets [10^9^/L] × ALT [U/L])

Laboratory values were obtained from fasting morning samples processed in the hospital’s central laboratory. Platelet counts were recorded in units of 10^9^/L; AST/ALT values below the analytical quantity limits were adjusted to the reportable lower limit before conversion. In accordance with current guidelines for older adults, we pre-classified participants with FIB-4 ≥ 2.0 as high risk and those with <2.0 as low risk. Because the FIB-4 score increases with age, it has been emphasized that a cut-off value of >2 is more appropriate for patients over 65 years of age. In the sensitivity analyses, traditional ranges (<1.30, 1.30–2.67, >2.67) were also reported for descriptive purposes [9].

### 2.5. Definition of Low Grip Strength

The EWGSOP2 criteria were used as the basis for identifying low grip strength. Handgrip strength was measured with a calibrated Jamar dynamometer (Performance Health, Warrenville, IL, USA). Following standardized positioning, participants performed three maximal trials with the dominant hand, each separated by ≥60 s; we recorded the mean value (kg). Consistent with population-validated Turkish cut-offs, <14 kg in women and <28 kg in men indicated low grip strength [16]. Functional status was characterized with (i) Basic Activities of Daily Living (Barthel Index, 0–100)**,** (ii) Lawton Instrumental ADLs (0–24 composite), (iii) Tinetti Performance-Oriented Mobility Assessment (POMA, 0–28), and (iv) the Timed Up-and-Go (TUG) test, recorded as the time (seconds) to stand from a standard chair, walk 3 m, turn, return, and sit. Geriatric syndromes were assessed per standard instruments: Mini Nutritional Assessment-Short Form (MNA-SF, 0–14) with malnutrition defined as ≤7 and at-risk 8–11; and Fried frailty phenotype (shrinking, exhaustion, slowness, weakness, low activity; classified as robust 0, pre-frail 1–2, frail ≥ 3). History of falls (past 12 months) and orthostatic hypotension (symptoms plus ≥ 20/10 mmHg drop) were recorded. Body mass index (BMI) was measured with a stadiometer and calibrated scale (Tanita Corporation of America, Inc., Arlington Heights, IL, US) on the same day.

### 2.6. Laboratory Assessments

Fasting venous blood was drawn between 08:00–10:00. The core panel comprised AST, ALT, hemoglobin, leukocyte and platelet counts, lipid profile (LDL-C, HDL-C, triglycerides), and estimated glomerular filtration rate (eGFR) computed by standard formulae. All assays were performed on manufacturer-recommended platforms with internal and external quality controls; inter-assay coefficients of variation for transaminases and platelet counts were within laboratory specifications.

### 2.7. Statistical Analysis

Data obtained in the study were analyzed statistically using IBM SPSS Statistics vn. 25.0 software (IBM Corp., Armonk, NY, USA). Continuous variables were stated as mean ± standard deviation (SD) values, and categorical variables as number (*n*) and percentage (%). In the comparisons between groups, Student’s *t*-test or the Mann–Whitney U-test was used for continuous data and the Chi-square test for categorical data. Relationships between the FIB-4 score and the clinical and laboratory parameters were evaluated with Pearson or Spearman correlation analysis. Logistic regression analysis was used to demonstrate the relationship between low grip strength and FIB-4 independent of confounding factors. Odds ratios of unadjusted, adjusted for demographic characteristics (Model 1), adjusted for Model 1 plus in terms of comorbidities and dementia (Model 2), and adjusted for Model 2 plus laboratory values (leukocyte count and total cholesterol levels) (Model 3) were calculated. In addition, it was tested whether the association between FIB-4 score and low grip strength varied by sex by including an interaction term (FIB-4 × sex) in the logistic regression models. Multivariate regression analysis was also performed for risk factors affecting high FIB-4 scores in dynapenic older adults. The covariate selection was determined as statistical significance. A value of *p* < 0.05 was accepted as statistically significant.

## 3. Results

A total of 537 older adults were stratified by FIB-4 into low (*n* = 372) and high (*n* = 165) groups. These two groups was initially compared. The mean age of the participants was statistically significantly higher in the high FIB-4 score group (78.60 ± 6.02 years) than in the low FIB-4 score group (75.60 ± 5.79 years) (*p* < 0.001). In terms of gender distribution the rate of females was significantly lower in the high FIB-4 score group (61.8%) than in the low FIB-4 score group (71.8%) (*p* = 0.022). The mean body mass index (BMI) value of the high FIB-4 score group was significantly lower than that of the low FIB-4 score group (25.47 ± 4.38 vs. 27.64 ± 5.69 kg/m^2^; *p* = 0.005).

In the evaluations of comorbidities and geriatric syndromes, the prevalence of diabetes mellitus was significantly lower in the high FIB-4 score group at 31.5% compared to 41.4% in the low FIB-4 score group (*p* = 0.030). Dementia (34.5% vs. 21.8%, *p* = 0.002) and malnutrition (40.2% vs. 31.7%, *p* = 0.048) were statistically significantly more common in the high FIB-4 score group. No statistically significant difference was determined between the groups in respect of hypertension, coronary artery disease, COPD, osteoporosis, and orthostatic hypotension.

In the high-FIB-4 group, leukocyte and platelet counts, as well as LDL-cholesterol and triglycerides, were significantly lower, whereas AST was significantly higher (all *p* ≤ 0.001); no significant differences were observed for hemoglobin, ALT, HDL-cholesterol, or eGFR.

**Figure 1:**
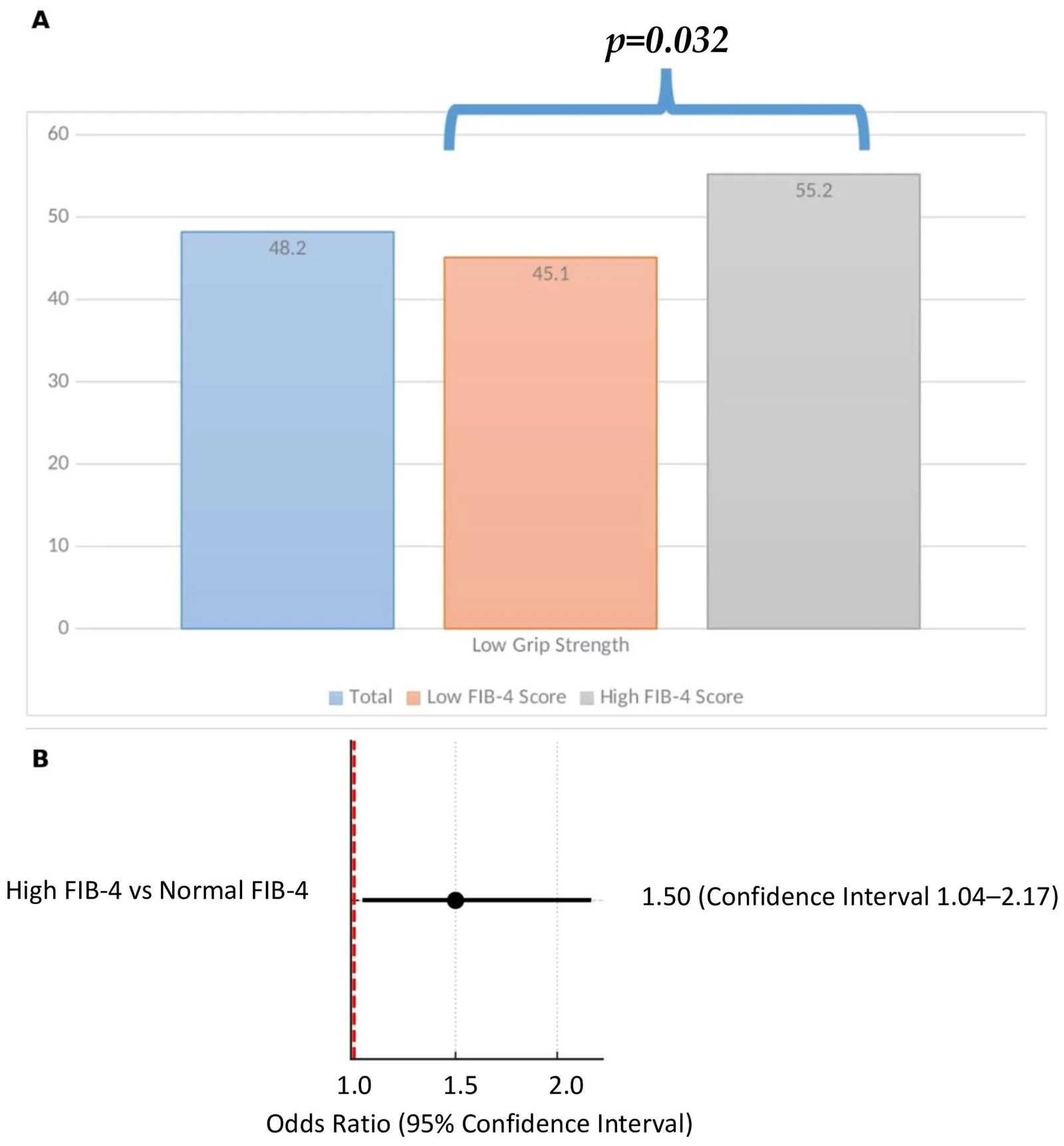
Comparison for the frequency of dynapenia according to FIB-4 status (**A**); Forest plot of the association between high FIB-4 and dynapenia. The black point denotes the odds ratio (OR), the horizontal whiskers indicate the 95% confidence interval, and the red dotted vertical line marks the null value (OR = 1) (**B**).

In the evaluation of the geriatric assessment parameters, the POMA scores (22.17 ± 6.40 vs. 23.40 ± 6.17, *p* = 0.017) and the instrumental ADLs scores (15.55 ± 6.17 vs. 16.95 ± 5.85, *p* = 0.002) were determined to be significantly lower in the high FIB-4 score group. No statistically significant difference was observed between the groups in respect of the ADLs scores and the TUG duration (Table 1). The frequency of low grip strength was found to be significantly higher in the high FIB-4 score group (*p* = 0.032). The odds of low grip strength were approximately 1.5 times higher in participants with elevated FIB-4 compared to those with low FIB-4 (OR 1.50, 95% CI 1.04–2.17) (Figure 1).

Interaction analysis demonstrated that sex significantly modified the relationship between FIB-4 score and low grip strength (*p* = 0.036). The odds of dynapenia increased with higher FIB-4 scores, and this effect was stronger in one sex (OR 1.19, 95% CI 1.05–2.36). In the correlation analyses performed according to gender, the FIB-4 score has a significant positive correlation with age and AST levels in both genders, and a significant negative correlation was determined with leukocyte and thrombocyte counts and with the functional parameters of hand grip strength, Tinetti-POMA, and Instrumental ADLs scores These correlations were observed to be more evident in females. The negative correlation between FIB-4 score and thrombocyte count was strong in both genders (females: r = −0.775, males: r = −0.717; *p* < 0.001 for both) (Table 2).

In univariate regression analysis, dynapenia is associated with high FIB-4 score in female older patients (Odds Ratio (OR): 1.52; 95% Confidence Interval (CI) 1.03–1.42; *p* = 0.043). However, there is no relationship between low muscle strength and fibrosis status in women regardless of confounding factors. Dynapenia is not associated with higher fibrosis status within older men (Table 3). In multivariate regression analysis, BMI values and leucocyte were associated with FIB-4 score within dynapenic older people (OR: 0.93 95% CI 0.86–0.99; *p* = 0.047 and OR: 1.00 95% CI 1.00–1.00; *p* = 0.004, respectively) (Table 3).

## 4. Discussion

The results of this study demonstrated a significant association between low grip strength and the FIB-4 score, which was used to predict liver fibrosis. The increased frequency of sarcopenia in individuals with a high FIB-4 score suggests that this non-invasive scoring system might reflect not only liver fibrosis, but also muscle power and gait–balance tests. Although these are preliminary results, this association appears to be more pronounced in women. However, significance is lost due to confounding factors. These findings indicate that the ageing processes of the liver and muscle tissue may share common pathophysiological pathways [17,18,19].

More than one pathophysiological mechanisms play a role in the development of malnutrition in patients with cirrhosis. Metabolic abnormalities, especially insufficient oral intake because of early fullness linked to moderate-severe acid, malabsorption, and decreased storage capacity with the metabolisation of nutrients by the liver are the most important factors, and these lead to loss of muscle mass and a worse prognosis by negatively affecting the energy and protein balance [20,21,22]. In this context, the capacity of the FIB-4 score to predict the risk of sarcopenia, is not limited to patients with liver disease, but may be significant for the general older adult population. This is supported by the current study findings that a lower BMI and geriatric syndromes such as malnutrition and dementia were seen more often in individuals with a high FIB-4 score. Previous studies have similarly reported significant relationships between sarcopenia and liver dysfunction [18,23,24,25].

The prognostic scope of the non-invasive fibrosis score FIB-4 for chronic liver disease follow-up has been shown to extend beyond hepatic outcomes with increasing evidence and to provide meaningful predictive value for extrahepatic clinical outcomes. Indeed, in a large community-based cohort, FIB-4 has been shown to be associated with an increased risk of mortality not only related to liver disease but also to all causes, cardiovascular disease, and even cancer-related mortality. This finding suggests that the simple biochemical components of FIB-4 (age, AST, ALT, platelets) may also reflect systemic disease burden [26].

In the prospective MyoVasc cohort of patients with heart failure, the FIB-4 score was found to be an independent predictor of all-cause mortality and showed significant correlations with indicators reflecting cardiac structure and dysfunction; this correlation was reported to be more pronounced in the non-MASLD subgroup. These observations indicate that FIB-4 acts as a biomarker that reflects not only hepatic fibrogenesis but also a combination of systemic processes such as congestion, low cardiac output, chronic inflammation, and endothelial dysfunction [27]. Similarly, the prognostic value of FIB-4 has been reported in various clinical conditions such as renal failure. In hemodialysis patients with chronic viral hepatitis, high FIB-4 levels have been independently associated with an increase in 5-year all-cause mortality. This suggests that FIB-4 should be interpreted as a comprehensive indicator of multi-organ interaction and systemic fragility rather than a “single disease score”. When translated into clinical practice, the use of FIB-4 as a screening tool in different disciplines may be considered [28]. It was primarily emphasized evidence from community-dwelling older adults, as this population directly reflects our research question. Studies in patients with heart failure, chronic kidney disease, or cirrhosis are mentioned only to illustrate potential systemic pathways of FIB-4, and these cohorts are not directly comparable to our sample.

Clinically it seems that FIB-4 can be used not only to discount liver fibrosis, but as an “early warning” screening parameter in respect of functional decline and potential sarcopenia in older adults. The relationships with TUG and grip strength in females especially with decreases in POMA and instrumental ADLs imply that an elevated FIB-4 score could be reflected in clinical performance. In this case, it would be rational to systematically screen nutrition, evaluate physical performance, and plan resistance exercises in older adults with a high FIB-4 score [29,30]. Although the TUG test is a widely used and sensitive tool for detecting mobility impairment, no significant difference was observed between FIB-4 groups in our cohort. This may reflect the large variability of TUG performance in very old adults, where comorbidities and heterogeneous functional reserves can attenuate between-group contrasts. In contrast, the POMA score, instrumental ADLs, and handgrip strength showed consistent associations with higher FIB-4, suggesting that this index may be more closely linked to multidimensional functional assessment rather than isolated gait speed or mobility performance.

The threshold value of FIB-4 used in this study was taken as ≥2.0 in accordance with the current EASL-EASD-EASO 2024 guidelines [3]. The findings obtained that this cutoff value could be effective in detecting both liver fibrosis and physical frailty in older adults. It has been reported in the literature that the FIB-4 score is not limited to being a non-invasive marker of liver fibrosis, but is also associated with cardiovascular mortality, frailty, and general morbidity [31,32]. This large older sample in geriatric practice, the FIB-4 score may not be simply a hepatic marker, but a holistic biomarker reflecting the systemic health status in older adults.

The number of older adults is increasing worldwide. Older adults constitute a significant portion of healthcare visits. It is known that comorbidities such as type 2 diabetes mellitus, hypertension, and cardiovascular disease increase with age [33]. Furthermore, the prevalence of MASLD also increases with age, and it has been reported that MASLD is a significant prognostic factor in advanced fibrosis [34]. On the other hand, it is well known that the frequency of age-related geriatric syndromes increases. These geriatric syndromes, particularly sarcopenia, frailty, and malnutrition, which contribute to significant morbidity and mortality, increase with age [35]. Therefore, it is noteworthy that the incidence of dynapenia in our study was higher in the group with higher FIB-4 scores, where age is a significant component. When considering the pathophysiological mechanisms of sarcopenia in older individuals, inflammation, mitochondrial dysfunction, and oxidative stress are prominent [36]. Considering that subclinical inflammation occurs with age, named inflammaging, and that inflammatory markers increase with age, this supports the association between sarcopenia and age. Other important components of FIB-4 score are ALT and AST. ALT is primarily a liver-specific enzyme, located in the hepatocyte cytosol and released with hepatocyte damage. AST, on the other hand, is not liver-specific. It is an enzyme found in many tissues, including muscle, heart, and lung. One study has shown that a high AST/ALT ratio is associated with sarcopenia. Accordingly, due to inflammation in skeletal muscle, enzymes related to skeletal muscle breakdown, including lactate dehydrogenase, creatine kinase, AST, and ALT, are released into circulation [37]. Similarly, inflammation can lead to muscle atrophy due to protein breakdown. Vitamin B6 serves as a cofactor in the synthesis and production of ALT [38]. In individuals with malnutrition, a geriatric syndrome closely associated with sarcopenia, dietary depletion of vitamin B6 will primarily reduce ALT and lead to an increase in the AST/ALT ratio. Furthermore, considering that atherosclerotic processes are also associated with a high AST/ALT ratio, sarcopenia will develop as a result of muscle atrophy in the extremities caused by atherosclerosis [37]. In other words, an increase in AST and ALT values in the FIB-4 score in favor of AST will increase the score, thus revealing the relationship in sarcopenic older individuals. Our study also demonstrated a relationship between dynapenia and FIB-4 scores in women, but not in men. Although this relationship disappeared due to confounding factors in women, an inverse correlation between FIB-4 scores and muscle strength was also demonstrated. Low grip strength emerged as the most consistent functional impairment associated with high FIB-4 in our study. Previous literature suggests that inflammaging, alterations in the AST/ALT ratio, and vitamin B6 depletion may contribute to muscle weakness through systemic inflammation and metabolic stress. While these mechanisms provide a plausible link between liver fibrosis risk and dynapenia, we did not measure inflammatory markers, sex hormones, or vitamin B6; therefore, these explanations should be considered hypothetical rather than empirically validated. Although a causal link cannot be established due to the cross-sectional nature of the study, it is known that the risk of sarcopenia and osteoporosis increases with a significant decrease in estradiol levels in postmenopausal women [39]. Estradiol, which is known to have a protective effect on hepatic steatosis, can also increase the risk of liver fibrosis [40]. In our cohort, the FIB-4 × sex interaction was statistically significant (*p* = 0.036; OR 1.19, 95% CI 1.05–2.36), indicating that the association between FIB-4 and sarcopenia differed by sex. While this finding supports the relevance of sex-specific mechanisms, such as hormonal changes, it should be emphasized that these pathways remain speculative as hormone levels were not measured in this study.

This study has several limitations. First, muscle mass was not assessed, and dynapenia was defined based on low grip strength, which may increase the risk of misclassification and limit diagnostic accuracy. Second, the cross-sectional design precludes causal inference, restricting interpretation to associations only. Third, participants were recruited from a tertiary care outpatient clinic, which may introduce selection bias and limit the generalizability of our findings to the broader community-dwelling older population. Taken together, these factors suggest that our results should be interpreted with caution and confirmed in larger, longitudinal, population-based studies with direct muscle mass assessment. A strong aspect of this study was that many geriatric parameters were evaluated in detail in a broad and well-defined elderly population. In addition, the correlation analyses performed according to gender subgroups allowed a more detailed evaluation of the results.

## 5. Conclusions

FIB-4 score appears to provide clinically meaningful, non-invasive information beyond hepatic outcomes in older adults. When interpreted with age-appropriate thresholds and used alongside brief functional and nutritional screening, FIB-4 may help identify patients at heightened risk for low grip strength and related adverse outcomes, supporting earlier, more integrated intervention in routine geriatric practice. FIB-4 may not be simply a hepatic marker, but a holistic biomarker. 

## Figures and Tables

**Table 1:** Comparison for demographic features, comorbidities, laboratory value, geriatric syndromes and comprehensive geriatric assessment parameters in terms of FIB-4 score status.

	Total Participants *n* = 537	Low FIB-4 Score *n* = 372	High FIB-4 Score *n* = 165	*p* Value
Demographic Features				
Age (mean ± SD)	76.52 ± 6.02	75.60 ± 5.79	78.60 ± 6.02	<0.001
Gender (female;%)	68.7	71.8	61.8	0.022
Education year	5.27 ± 3.74	5.29 ± 3.82	5.23 ± 3.56	0.909
Marital status (marriage;%)	55.1	56.7	51.5	0.200
BMI (kg/m^2^)	26.90 ± 5.37	27.64 ± 5.69	25.47 ± 4.38	0.005
Comorbidities and Geriatric Syndromes (%)				
Hypertension	72.6	74.5	68.5	0.152
Coronary Heart Disease	21.0	20.7	21.8	0.769
Peripheral Artery Disease	5.8	6.5	4.2	0.311
Diabetes Mellitus	38.4	41.4	31.5	0.030
COPD	5.8	4.8	7.9	0.163
Osteoporosis	25.5	26.1	24.2	0.653
Dementia	25.7	21.8	34.5	0.002
Orthostatic Hypotension	43.8	44.9	41.1	0.426
Malnutrition	34.3	31.7	40.2	0.048
Polypharmacy (≥5 medication use)	67.0	68.3	64.2	0.359
Frailty	40.0	39.0	42.4	0.452
Laboratory Findings (mean ± SD)				
Hemoglobin (g/dL)	12.65 ± 1.67	12.63 ± 1.63	12.68 ± 1.75	0.658
Leucocyte count (×10^3^/µL)	7.25 ± 2.31	7.57 ± 2.35	6.52 ± 2.04	<0.001
Platelet count (×10^3^)	256.93 ± 94.82	285.60 ± 97.14	191.33 ± 42.95	<0.001
ALT (IU/L)	17.34 ± 14.57	16.97 ± 10.49	18.16 ± 21.06	0.258
AST (IU/L)	21.21 ± 11.67	19.25 ± 8.35	25.62 ± 16.08	<0.001
eGFR (mL/min/1.73 m^2^)	65.86 ± 18.98	66.43 ± 19.14	64.57 ± 18.59	0.246
LDL-cholesterol (mg/dL)	125.50 ± 37.95	128.88 ± 38.96	117.83 ± 34.47	0.001
HDL-cholesterol (mg/dL)	57.02 ± 14.02	57.13 ± 14.16	56.77 ± 13.73	0.958
Triglyceride (mg/dL)	140.58 ± 108.27	147.72 ± 122.58	124.33 ± 62.18	0.001
Comprehensive Geriatric Assessment Parameters				
Tinetti POMA score	23.02 ± 6.26	23.40 ± 6.17	22.17 ± 6.40	0.017
Timed up and go test duration (s)	22.16 ± 20.03	21.64 ± 19.66	23.36 ± 20.85	0.125
Basic ADLs	85.30 ± 16.46	85.82 ± 16.10	84.13 ± 17.24	0.164
Instrumental ADLs	16.52 ± 5.98	16.95 ± 5.85	15.55 ± 6.17	0.002

ADLs: activities daily of living; ALT: alanine aminotransferase; AST: aspartate aminotransferase; BMI: body mass index; COPD: chronic obstructive pulmonary disease; eGFR: estimated glomerular filtration rate; HDL: high density lipoprotein; LDL: low density lipoprotein; POMA: performance-oriented mobility assessment; SD: standard deviation.

**Table 2:** Correlation between FIB-4 score and age, medication use, comprehensive geriatric assessment parameters, laboratory findings and muscle strength within sex groups.

	Female Older Adults		Male Older Adults	
	Correlation Coefficient (r)	*p* Value	Correlation Coefficient (r)	*p* Value
Age	0.277	<0.001	0.230	0.003
BMI (kg/m^2^)	−0.101	0.204	−0.052	0.632
Medicine number	−0.027	0.601	−0.063	0.421
Leucocyte count (×10^3^/µL)	−0.345	<0.001	−0.256	0.001
Platelet count (×10^3^)	−0.775	<0.001	−0.717	<0.001
LDL-cholesterol (mg/dL)	−0.101	0.057	−0.113	0.149
Triglyceride (mg/dL)	−0.204	<0.001	−0.088	0.263
ALT (IU/L)	−0.036	0.496	−0.157	0.042
AST (IU/L)	0.420	<0.001	0.421	<0.001
MNA-SF score	−0.096	0.066	−0.106	0.171
POMA score	−0.167	0.001	−0.103	0.188
TUG duration	0.121	0.022	0.089	0.257
FRIED score	0.102	0.052	0.079	0.310
Basic ADLs	−0.102	0.050	−0.044	0.569
Instrumental ADLs	−0.157	0.003	−0.147	0.057
Handgrip strength (kg)	−0.111	0.035	−0.125	0.109

ADLs: activities daily of living; ALT: alanine aminotransferase; AST: aspartate aminotransferase; BMI: body mass index; LDL: low density lipoprotein; POMA: performance-oriented mobility assessment; TUG: timed up and go test.

**Table 3:** Univariate analysis between low grip strength and FIB-4 score status within gender subgroups, and multivariable analysis high-FIB-4 status within dynapenia group.

		High FIB-4 Score	OR	95% CI (Min–Max)	*p* Value
Univariate analysis	Female	Dynapenia			
		Unadjusted	1.52	1.03–1.42	0.043
		Model 1	1.19	0.50–2.80	0.684
		Model 2	1.31	0.54–3.19	0.543
		Model 3	1.12	0.42–2.98	0.816
	Male	Dynapenia			
		Unadjusted	1.31	0.70–2.47	0.392
		Model 1	0.97	0.28–3.35	0.962
		Model 2	0.88	0.23–3.33	0.852
		Model 3	0.60	0.14–2.64	0.508
Multivariable analysis	Variables	Gender	1.05	0.50–2.21	0.891
		BMI	0.93	0.86–0.99	0.047
		DM	0.92	0.49–1.91	0.926
		Dementia	1.23	0.49–3.03	0.652
		COPD	2.68	0.69–10.32	0.151
		Malnutrition	1.17	0.55–2.49	0.668
		Leucocyte count	1.00	1.00–1.00	0.004
		Total cholesterol	0.99	0.98–1.00	0.165
		Instrumental DLs	1.04	0.94–1.14	0.386
		POMA score	0.99	0.92–1.06	0.884

BMI: body mass index; CI: confidence interval; COPD: chronic obstructive pulmonary disease; DM: diabetes mellitus; FIB-4: fibrosis-4; OR: odds ratio; POMA: performance-oriented mobility assessment. Model 1—Adjusted for body mass index (BMI) and marital status; Model 2—Adjusted Model 1 plus the presence comorbidities including diabetes mellitus and dementia; Model 3—Adjusted Model 2 plus laboratory findings.

## Data Availability

The original contributions presented in this study are included in the article. Further inquiries can be directed to the corresponding author.

## Abbreviations

The following abbreviations are used in this manuscript:

FIB-4	The fibrosis-4
AST	Aspartate transaminase
ALT	Alanine transaminase
MASLD	Metabolic dysfunction-associated steatotic liver disease
COPD	Chronic Obstructive Pulmonary Disease
ADLs	Activities of Daily Living
POMA	Performance-Oriented Mobility Assessment
TUG	Timed Up and Go
EWGSOP	European Working Group on Sarcopenia in Older People
MNA-SF	Mini Nutritional Assessment-Short Form
GFR	Glomerular filtration rate
